# Experiential Learning in a Gamified Pharmacy Simulation: A Qualitative Exploration Guided by Semantic Analysis

**DOI:** 10.3390/pharmacy9020081

**Published:** 2021-04-15

**Authors:** Denise L. Hope, Gary D. Rogers, Gary D. Grant, Michelle A. King

**Affiliations:** 1 School of Pharmacy and Medical Sciences, Griffith University, Gold Coast, QLD 4222, Australia; g.grant@griffith.edu.au (G.D.G.); michelle.a.king@griffith.edu.au (M.A.K.); 2 School of Medicine, Deakin University, Geelong, VIC 3217, Australia; g.rogers@deakin.edu.au

**Keywords:** pharmacy education, gamification, simulation, experiential learning, active learning

## Abstract

Experiential learning is an important component of pharmacist education and is primarily achieved through supervised placement or simulation. This study explored senior pharmacy students’ experiential learning in an extended, immersive, gamified simulation, conducted as a capstone learning activity toward the end of their final year of study, consolidating all prior learning and preparing students for intern practice. The simulation aimed to enhance student confidence, competence and collaboration. The three-week activity involved student teams competitively managing simulated pharmacies, assuming the role of pharmacists to complete all scaffolded assessments, including dispensing prescriptions, clinical cases, verbal counselling, simulated patient cases, interprofessional collaboration, and assignments. Assessments were marked continuously, with consequences of practice acknowledged through gain or loss of ‘patients’ for the pharmacy. From 2016 to 2018, 123 students completed multiple individual reflective journals (*n* = 733). Reflective journals were analyzed to explore the student experience, using a mixed methods approach. Initial Leximancer® 4.51 semantic analysis guided thematic analysis, conducted in NVivo® 12. The major themes that emerged were *teamwork*, *patient-centeredness*, *medicines provision*, *future practice*, and the *learning experience*. Student participants reported an intense and emotional experience in the gamified simulation, with many students revealing transformation in their skills, behaviors and attitudes over its duration.

## 1. Introduction

Experiential learning in its simplest form is learning by doing. Kolb’s seminal (1984) research and his model of the experiential learning cycle detail the importance of reflecting on an experience to create meaning, which leads to further action and active experimentation in being open to, and valuing, the experience [1]. According to Kolb’s theory, the stimulus for developing new ideas is exposure to new experiences [1,2]. Experience is transformed into knowledge [3] and helps students to develop values, skills, and attitudes [4,5] that are crucial to the development of pharmacist competence and the specific competencies required of the pharmacy graduate [6]. Experiential education is an important component of contemporary pharmacist, health professional, and interprofessional education that is primarily achieved through supervised placement or simulation [5,7,8,9,10].

An immersive, extended, gamified simulation was implemented as a capstone activity for final year students of new Bachelor and Master of Pharmacy programs at Griffith University, Australia. The four-year undergraduate Bachelor of Pharmacy was targeted at school leavers, and the two-year intensive post-graduate Master of Pharmacy program was targeted at candidates with a prior science or health science degree. Both programs lead to provisional registration with the Pharmacy Board of Australia, which then involves an internship year of supervised practice prior to general registration as a pharmacist in Australia. The capstone nature of the simulation consolidated student knowledge [11,12] by refreshing prior learning and iteratively revisiting previous topics, aligning with the concept of a spiral curriculum [13]. The gamified simulation, or serious game, was based on the Pharmacy Game developed at the University of Groningen [14,15], The Netherlands, which has been adopted by a small consortium of international universities. 

The Australian Pharmacy Game involved student teams competitively managing simulated pharmacies for three weeks, toward the end of their degree program. All final year Bachelor and Master of Pharmacy students participated. The simulation typically involved six to eight student teams, each comprising five to eight students, completing continuous pharmacy-related tasks. The initiative utilized simulated patients and aimed to provide an authentic learning experience in which students assumed the role of autonomous pharmacists in all decision-making, interprofessional contact, and patient consultations, to develop their professional and collaborative skills [16]. Gamification of the simulation introduced story, scoring, and competition, which enhanced participant motivation and engagement [17], while delivering consequences for all clinical and professional decisions and actions through continuous scoring of the regular and scaffolded assessments [18], marked by pharmacist academics and practicing pharmacists. Activities in the simulation included daily prescription dispensing; clinical and patient cases; telephone consultations with patients, carers, or prescribers; in-person and recorded medication counselling and assignments, such as preparation of adverse drug reaction or drug use evaluation reports. 

During the simulation, student teams conducted regular debriefing sessions as pharmacy staff meetings and completed multiple individual written reflections. Debriefing during simulation is vital to help participants make sense of events, feelings and outcomes, and the debriefing should encourage reflection [19]. Reflection is an essential component of experiential learning [1] and critical reflection is recognized as an important skill for deep understanding and insight that informs professional development and expertise [16,20,21]. Students’ critical reflection facilitates construction of professional identity and contributes to their development as self-directed and lifelong learners [20,22].

While the gamified pharmacy simulation educational approach has been used by a small number of universities for many years [14,15,23,24,25,26], there is a dearth of research detailing the influence on students’ experience of learning from such an intensive and emotionally impactful activity. This research aimed to evaluate senior pharmacy students’ experiential learning during participation in an extended, immersive gamified simulation. This was achieved through qualitative thematic exploration of student reflective journals, guided by a quantitative semantic analysis.

## 2. Methods

A mixed methods approach was adopted to conduct a qualitative thematic analysis of students’ reflective journal text, guided by a quantitative semantic analysis. Mixed methods research may facilitate a more comprehensive understanding of an issue and has been suggested as a suitable approach to provide a more accurate and complete review of educational experiences and outcomes in pharmacy education [27].

Senior pharmacy students of an Australian university were invited to participate in the research toward the end of their respective Bachelor or Master of Pharmacy programs, during the first three annual iterations of the gamified simulation. During the simulation each student completed multiple reflective journals following team debriefing. Students were provided advance guidance on the conduct of the debriefings, and supervisory staff visited with teams during the debriefing sessions wherever possible. Students had received previous instruction on writing critical reflections, as they had been assessed on reflections in multiple subjects. Journals were marked by external assessors with team scores reported to students. Participants provided written consent for the primary researcher (D.L.H.) to access their textual reflective journals, after the gamified simulation was finished and any formal student–lecturer interaction had ceased. Institutional ethical approval was obtained from the Griffith University Human Research Ethics Committee (2016/594).

The reflective journals were contemporaneous records of students’ participation in, and experience of, the gamified simulation. Across the three-week duration of the simulation multiple reflections were completed and uploaded as textual files to the university’s Learning Management System and subsequently downloaded with consent by the primary researcher (D.L.H.). Downloaded files were deidentified by coding with a unique identifier and removing proper names from in-text comments that identified other students or staff. During the simulation, student welfare was monitored by a staff member, unrelated to the research, who regularly checked reflections for risk management purposes and provided interventions where required. On rare occasions, students of concern were contacted privately by staff to assess welfare and provide support and referral, if required.

Semantic analysis was employed to guide and provide confirmatory analysis of the large quantity of reflective journals in this project. Leximancer® 4.51 is a software solution for qualitative data analysis and was used to conduct the semantic analysis, as it is well suited to processing large volumes of text to automatically identify concepts and textual interrelationships [28]. Automatic semantic analysis of text is efficient and reduces the subjectivity of human analysis [28]. Leximancer performs a type of content analysis that discovers and extracts thesaurus-based concepts from text data. It codes the concepts according to the thesaurus that has been built, automatically detecting concepts and clustering them into themes through determination of semantic frequency and connectivity [29]. The concept co-occurrence data revealed informs the generation of a concept map [30]. Leximancer’s identification of themes employs the hierarchy of concept connectedness, in which a highly connected concept is a parent of a thematic region and is used to characterize that region [29].

Thematic analysis was conducted using NVivo® 12 software to further examine and code the data, guided by the concepts and themes of the semantic analysis. The advantage of using a combination of Leximancer and NVivo in the analytic process is that the data set can be viewed from different perspectives and the results integrated providing a richer interpretation of a large data set [28,31]. The primary researcher (D.L.H.) used NVivo to code data according to the themes generated in Leximancer. Themes and coding were confirmed by another member of the research team (M.A.K.). Exemplar student textual quotes were copied into a Microsoft® Excel® 2008 spreadsheet, divided into sheets for each Leximancer-identified theme, providing a framework for the thematic analysis [32]. The combined results enable rich exploration and meaning-making of the data.

## 3. Results

From 2016 to 2018, 123 senior pharmacy students completed 733 textual reflective journals during the gamified simulation. Reflective journals were approximately 300 to 500 words in length. All students participating in the gamified simulation consented to the reflective journal research; two-thirds were female (Table 1).

### 3.1. Semantic Analysis

**Figure 1:**
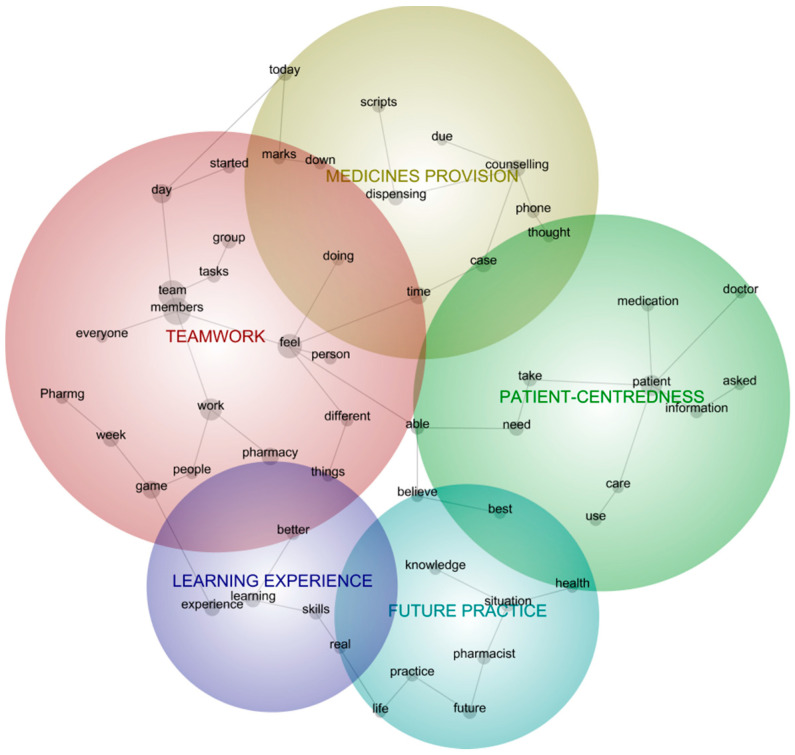
Leximancer thematic and concept map.

The 733 de-identified reflective journals were selected as input documents for the Leximancer analysis. Text processing settings allowed for identification of name-like concepts and merged word variants. Concept seed settings were set to default. When concept seeds were generated, two text concept merges were manually conducted: ‘feel’ with ‘felt’, and ‘today’ with ‘Today’. When the concept map was generated, 11 themes were initially presented. The concept map was manually reclustered 15 times, until the generated themes were stable, and totaled six. All component concepts in the map were made visible (100%) and the map rotation was set to 151 degrees, to assure their visibility. The theme size was then adjusted to 54%, which reduced visible themes to the five most important ones identified. The Leximancer-identified theme that was eliminated in the visibility adjustment was *today*, which was generated due to the ubiquity of the word ‘today’ in journals but was considered less important to analysis and understanding of the student experience. The automatically generated themes were *team*, *counselling*, *patient*, *future*, and *experience*. The primary researcher (D.L.H.) renamed these for clarity and to align with the pharmacy education context, to *teamwork*, *medicines provision*, *patient-centeredness*, *future practice* and *learning experience*. Figure 1 diagrammatically presents the results of the semantic analysis conducted in Leximancer.

The heat mapping of the concept map reveals themes in descending order of importance where importance is determined by semantic frequency and co-occurrence of words and concepts in the concept map. Warmer colors (red, orange) indicate the most relevant concepts, whereas cooler colors (blue, green) denote the least relevant [30]. Leximancer defines relevance as the number of occurrences of the concept as a proportion of the most frequently occurring concept. The most important theme identified was *teamwork* (red), with component concepts related to daily collaborations. The theme of *medicines provision* (yellow) identified students’ focus on case work, including dispensing and counselling, whereas *patient-centeredness* (green) was about providing care and information to the patient. *Future practice* (blue-green) identified students’ translation of the experiential learning to their future practice and development of real-life skills and the theme of *learning experience* (purple) covered concepts about the learning experience and application of real-life skills. The main concepts identified in semantic analysis are recorded by theme in Table 2.

### 3.2. Thematic Analysis

The five primary themes identified during sematic analysis were confirmed during thematic analysis. The themes were complex and often interrelated, as indicated by the supporting exemplar quotes presented narratively. Additional quotes are summarized in Supplementary Table S1.

#### 3.2.1. Teamwork

The theme of teamwork was the most dominant in the semantic analysis and this was reinforced by the thematic analysis. The majority of students’ journals reflected on various aspects of working in a team and confirmed that three weeks of constant collaboration and teamwork was educationally, professionally, and emotionally impactful on their experience:

*I have also experienced the power of strong communication and teamwork, and how much people are willing to support you if you just ask for it.*

(P141, 2018)

*I have really learnt a lot about teamwork over the past few weeks. Having access to colleagues to help brainstorm and solve problems really is an invaluable asset, and one that I think many people overlook.*

(P147, 2018)

The competitive nature of the gamified simulation, and assessment of all tasks, was reflected upon by many students, who reported the stress or pressure of not wanting to let their team down:

*Being a part of a group and being responsible for a team mark is definitely a bizarre kind of pressure. A couple of the people in my group being the highest achievers of the class, I had to make sure that I never lost any point for them.*

(P003, 2016)

Student anxiety and apprehension about the simulation was more evident at the outset but seemed to improve or resolve for most, as the simulation progressed:

*I was concerned about time management within the group, group member participation, and the marking per activity. However, even only after the first day all of those concerns have disappeared.*

(P021, 2016)

*At first I was very scared and anxious that I would let the team down due to my lack of real work experience... As the game progressed I became less anxious and my confidence in my abilities grew...*

(P133, 2018)

As the simulation progressed, many students reported a shift in mindset, away from the pressure of the marking to recognition of the value of the experience:

*After witnessing how driven and competitive each team is by the PharmG scoreboard, I am starting to feel we are all placing too much value on how many patients we are accumulating instead of what we are gaining from the experience.*

(P005, 2016)

*But really, the scores don’t mean anything to me. I’m just thankful I had such an amazing team that was supportive and we all tackled the game as a group, there was no leader we shared all of the jobs and gave help when it was needed.*

(P138, 2018)

Positive aspects of teamwork reflected upon included both learning from others and helping to support the learning of others:

*I have found that through teaching and explaining things to my teammates that I am in fact learning things and expanding my knowledge on cases and learning from other’s opinions and experiences.*

(P073, 2017)

The teamwork experience also appeared to provide students with insights into themselves and others:

*I think I need to understand that everyone has their individual way of learning and they may not appreciate my feedback the way I would. … For future feedback I need to make sure I include positive points as well as constructive feedback to make sure my criticism doesn′t come across too negative.*

(P008, 2016)

Several students also journaled about learning to lead, because of the teamwork:

*Overall, as frustrating as this may have seemed to me, I have found this experience invaluable, and I liken my role in these events to that of a manager, where I have had to settle disputes between employees to allow for work to be completed unhindered.*

(P128, 2018)

As a consequence of the teamwork, some students reflected on power struggles between team members and managing interpersonal conflict:

*The scenarios included many ‘walk-in’ customers with specific queries and product requests. ... It became almost a struggle for power with questions being asked over and over again and the conversation going around in circles.*

(P093, 2017)

*Proactively dealing with conflict within a workplace environment is not a strength of mine, however, during the first couple of days I was left with no alternative but to speak up and give an opinion, which has impacted my self-confidence in a profound and positive way.*

(P085, 2017)

#### 3.2.2. Medicines Provision

Medicines provision was a dominant theme to emerge from the data, as almost all students reflected on the tasks and activities of the simulation, which focused on safe and appropriate provision of medicines. Reflections on dispensing tasks and provision of counselling were often connected to real-world practice:

*We quickly sorted ourselves and found jobs for everyone, mine was dispensing and counselling a medication to be collected by the patient later in the day. This made me feel as if I actually was in a real pharmacy with real patients.*

(P004, 2016)

Students often reported the pressure of the time, tasks, and workload in their daily activities, but many linked this to real-world pharmacy practice:

*This has made me realize I need to prioritize my counselling points for each script. In practice, not dispensing a patient’s prescription in time is not good for the business and my reputation as a pharmacist.*

(P023, 2016)

Reflections on the dispensing and counselling tasks at times revealed the impact of students learning from their own or others’ errors:

Following a case designed to require students to amend a third-party medicine request without breaching patient privacy and confidentiality:

*Something that I couldn′t stop thinking about today was the fact that I disclosed a patient’s confidential information, and I chastise myself over it... I couldn’t even believe how foolish I was to have ignored the warning bells inside my head.*

(P018, 2016)

The variety of tasks and topics students were exposed to during the simulation was often positively reflected upon:

*I liked that in the game there was plenty of cases based on things that we wouldn’t always think about. For example, questions from patients about what drugs they can use in certain sports or vaccination enquires, this made me use a variety of resources that I don’t use often.*

(P125, 2018)

#### 3.2.3. Patient-Centeredness

Reflections on the patient interactions often described how students learned to focus on the patient as their main priority:

*In all my future counsellings, I will always consider the patient as an equally effective member in the healthcare team. After all, the patient is the centre of care.*

(P004, 2016)

Some students reported insights that they experienced in dealing with simulated patients that may have presented challenges to communication and provision of care:

*It was* [a] *realistic learning experience to deal with tough clients from different backgrounds.*

(P140, 2018)

Case with a deaf patient:

*This experienced caught me off guard and has challenged the way I will deal with them in the future. To prepare for similar consultations, I should develop a process to assist me in evaluating the impact an impairment/disability may have on a person’s ability to take in the counselling/advice I am providing.*

(P079, 2017)

Some of the simulated patient interactions reportedly had an emotional impact on the students, particularly those that involved the death of a patient:

*For last week, when the elderly lady had died … I felt sad, that it was a case of penicillin related allergies, and that the other pharmacists hadn′t picked up on this.*

(P166, 2018)

*...one of the highlights of the game that really touched me and changed my level of thinking was when we had an actor pretending his close family member passed away. That made me very upset, it made me think about how possible it is to deal with that kind of customers in the pharmacy during our everyday lives.*

(P075, 2017)

Many students reflected on holistic patient care and how their perspectives changed during their experience of the gamified simulation:

*My understanding of patient centered care has grown significantly during the game … I believe every case should be done differently, it is the only way to provide holistic care.*

(P026, 2016)

#### 3.2.4. Future Practice

Many students considered the influence of the gamified experience on their future practice as pharmacists:

*The problems we have had, reinforces to me the importance and value of communication with your staff members in the workplace to achieve positive outcomes. ... Even though this is called the game, I can see how it is preparing me to become a diligent Pharmacist.*

(P151, 2018)

Interprofessional practice was an attribute of the simulation often positively reflected upon in student journals, and some also reported interprofessional experiences as learning opportunities:

*So far, this activity has been a great learning experience and has positively changed my attitude towards collaborating with other health professionals. I have learnt to be more confident, calm and professional during communication between another health profession/students and also between a customer under highly stressful situations.*

(P089, 2017)

*... one particular incident ... that taught me a lot, ... would be when I was ill prepared for my interaction with a prescriber. I learned that I should be far more prepared and armed with knowledge/suggestions or secondary options before calling a prescriber and taking up some of their valuable time.*

(P139, 2018)

Consequences of actions and behaviors in the simulation were reflected upon in the context of future practice:

*Even though it wasn′t me that made such a careless error, I realize that in practice nobody will be checking my work, and one small mistake can have devastating consequences not only for my career in pharmacy, but a patient’s health and wellbeing.*

(P133, 2018)

#### 3.2.5. Learning Experience

Students reflecting on their overall learning experience during the gamified simulation described the emotion and intensity of the experience:

*I felt like I have exercised all my emotions during this Pharm G experience, but was also grateful for the experience. I have been shaped in all areas that I would not have had an opportunity to work on if I was practicing in a real pharmacy.*

(P024, 2016)

*We have come to almost the end of one of if not the most intense learning experience of the pharmacy degree. Something that I will gladly say has been an invaluable experience…*

(P002, 2016)

The safety of the simulation and authenticity of the pharmacy environment were discussed:

*Despite the rough start, I still see the value in such a simulation, as there is no other environment where you can gain real-life experience in a way where there is no risk of compromising patient safety.*

(P088, 2017)

One of the most frequent criticisms reported in reflective journals was the lack of overt feedback in the simulation. One student acknowledged that the approach was more reflective of practice:

*Although we were getting assessed on everything we did, we didn’t receive feedback on everything. That was somewhat stressful … we do not often get feedback for what we do out in the real world. We have to learn from how people react to our actions and sometimes feedback from colleagues.*

(P134, 2018)

Many students reported on transformations they made in their own learning and outlook, as a result of their experience:

*I think that this activity made me a more professional and independent learner because even if we work as a team, I still have to make evidence-based decisions for the scenarios I was handling.*

(P099, 2017)

*Actually, sometimes some mistakes are a good opportunity to grow stronger and to be more resilient.*

(P168, 2018)

*Though I have learnt a lot about myself and my other teammates. This game has changed my views on how a team can work together and how to manage a task load. It’s been a challenging experience but I’m glad I had it.*

(P019, 2016)

## 4. Discussion

Students’ reflections on their experience in the immersive, gamified simulation confirmed that the activity supported teamwork and collaborative learning. Valuable insights into pharmacy students’ experiential learning in the simulation and its impact on professional identity and future practice were revealed. This appears to be the first study to expose the impact on, and emotions of, students undergoing this type of intensive learning activity, which then provides valuable feedback to educators on many of the simulation approaches, processes and component activities conducted within variations of the Pharmacy Game. 

Several Pharmacy Game participant universities have published on their respective gamified simulations, however the detail of the student experience was limited. Early research from the University of Groningen used student surveys to report briefly that participants enjoyed the game and felt that it supported their integration of knowledge and skills in preparation for intern practice [14,23]. Subsequent research used official evaluation reports, which conveyed that students in their Pharmacy Game felt motivated to learn but lacked feedback on their performances [15]. This mirrors our findings that students valued such aspects of the simulation but were frustrated with the lack of direct feedback, although some recognized the latter emulated the more subtle feedback mechanisms anticipated in professional pharmacist practice. Researchers from Utrecht University, The Netherlands, reported on the use of the Pharmacy Game GIMMICS^®^, in a competency-based curriculum, to provide authentic pharmacy practice simulation under safe, supervised conditions [25,33], and to prepare students for internship [26], but did not present student outcome data. Conference abstracts from the University of Nottingham, United Kingdom, focused on positive aspects of students’ teamwork [34] and experiential learning [35] in their Pharmacy Game offering, the Pharmacy Leadership and Management simulation. This Australian study positively builds on previous research and provides the student voice to reveal insights about experiential learning in this type of gamified simulation.

The dominant theme to emerge from student participants’ reflective journals was teamwork, with impacts of teamwork conveying both positive and negative emotions. Students reflected on learning to navigate team dynamics, managing emotions and conflict within a team and also the positive collaborations that resulted from teamwork. This is an important outcome, critically aligned with the learning objectives of the activity [36], as building cooperative and respectful relationships with the pharmacy team and with other health professionals is a recognized competency for pharmacists [6,37]. While uncertainty and the focus on marks were pervasive during the early days and weeks of the simulation, with students not wanting to let their teams down, the focus seemed to shift away from the concern with marks to a more professional one as students settled into their pharmacist roles. This transformation allowed for the establishment of professional values and identity [16]. By the close of the simulation, most participants reflected positively on teamwork, including improved interpersonal communication, collaboration, conflict management, and overall satisfaction with their perceptions of the experience.

Reflections related to the theme of medicines provision often involved recall of actions and behaviors conducted during the experience but there was a distinct link to real-world and future practice, emphasizing the importance of simulation of these tasks in a safe environment [38,39]. The tasks related to medicines provision also appeared to give students feedback on their own management of time, task and workload, and importantly, enabled learning from errors, which is a recognized advantage of simulation [40,41,42]. Students having the autonomy to make mistakes, acknowledge their limits and learn from errors is an important aspect of self-determination theory [43,44,45,46] and aligns with the objectives of simulation [47] and this gamified simulation in particular.

Patient-centeredness was a theme that emerged from the data analysis and focuses on the interests of the patient as the first priority, recognizing and tailoring care to the patient’s individual preferences [48,49]. Students reflected on their simulated patient interactions and often reported transformations in their awareness and approaches to patients, due to their experiences. The concept of holistic patient care was often reflected upon, which aligns with research suggesting that critical reflection can enhance the holistic approach to care [22]. The simulation provided students with opportunities for inter-professionalism [7] and enabled focus on the whole person, to develop their interpersonal patient relationships. It positively rewarded optimal patient outcomes, which is conducive to the development of patient-centeredness [49]. The authenticity of the activity, including use of simulated patients, added realism and impact to the patient interactions, as students were able to apply their skills and knowledge as in real-world practice [5,16,50]. 

The theme of future practice linked to many elements of the other themes, as students reflected on teamwork, medicines provision, and patient-centeredness in the context of their future roles as health professionals. Reflections provided important insights into students’ translation of their learning in the simulation to their vision of their future professional practice, sometimes describing how they ideally see themselves practicing in the future. The authentic learning environment supported enhancement of professional behaviors and identity [16]. The opportunities for inter-professionalism during the simulation were very impactful, with many students describing how they improved their confidence and competence to converse and collaborate with prescribers. This is an important outcome from the simulation and aligns well with professional expectations of pharmacists as members of the multidisciplinary team [6,7,37]. 

The theme of learning experience that emerged from the data encompassed student reflections on the overall experience, which were often emotional and described the activity as intense, exhausting but also rewarding and transformative. A recurrent concept negatively reflected upon was the lack of overt feedback, which is consistent with findings from similar gamified simulations [15]. Overall, students valued the plethora of learning opportunities and safety within the simulation, being able to apply their knowledge, practice their skills and learn from their errors, all in an environment without risk of patient harm [38,39], which are essential aspects of healthcare simulation and a learning objective of this gamified simulation. 

This study revealed the synergistic advantage of conducting complementary semantic and thematic analyses, using both Leximancer and NVivo. The thematic analysis identified a degree of commonality between themes that may not have been so clearly evident from the semantic analysis outputs, such as the commonalities in reflections on future-practice and patient-centered care. While the Leximancer analysis related to medicines provision frequently detected occurrence and co-occurrence of concepts about dispensing and counselling of medicines, the thematic analysis revealed that these reflections were less emotional than those of themes like teamwork or future practice. That said, if the thematic analysis had been conducted without the guidance of the initial semantic analysis, it might have been easy to overlook the reflections on medicines provision as perfunctory. However, they provide important insights into the students’ experiential learning as it is in those pharmacists’ tasks that the simulation and modeling of future practice occurs. 

The strengths of this research include the large volume of data that was available for analysis, collected from students in two pharmacy degree programs over three years. In addition, the mixed methods approach to analysis helped to limit researcher bias in thematic analysis coding and interpretation and allowed for rich exploration and triangulation of data. A limitation of the research is that analysis relied solely on students’ reflective journals. It was assumed that their content was an honest reflection on the student experience, but the veracity of each reflective journal cannot be guaranteed [51].

## 5. Conclusions

Participation in an extended gamified simulation allowed students to adopt the pharmacist’s role in a safe learning environment. The authentic nature of the simulation enabled them to develop collaboration, patient-centeredness, confidence, and professional identity. While students reported an intense and emotional experience in the gamified simulation, many reflected on the positive transformation in their skills, behaviors and attitudes over its duration.

## Figures and Tables

**Table 1:** Participants and reflective journals by year of participation.

		2016		2017		2018		Total	
		*n*	(%)	*n*	(%)	*n*	(%)	*n*	(%)
Participants	Male	8	(29.6)	15	(31.9)	17	(34.7)	40	(32.5)
	Female	19	(70.4)	32	(68.1)	32	(65.3)	83	(67.5)
	Total	27		47		49		123	
Journals	Male	96	(29.8)	54	(32.0)	75	(31.0)	225	(30.7)
	Female	226	(70.2)	115	(68.0)	167	(69.0)	508	(69.3)
	Total	322		169		242		733	

**Table 2:** Themes and concepts identified in Leximancer.

Theme	Concepts
Teamwork	team, members, work, tasks, group, day, everyone, started, doing, time, person, pharmacy, different, people, game, week, PharmG ^1^
Medicines Provision	case, counselling, thought, felt, due, down, time, phone, doing, dispensing, scripts, today, marks
Patient-Centeredness	patient, medication, information, doctor, care, asked, use, best, health, situation, take, need, believe, able, things
Future Practice	pharmacist, knowledge, future, practice, real, life, health, situation
Learning Experience	learning, experience, feel, better, real, skills, people

^1^ PharmG is the name for the Pharmacy Game at Griffith University.

## Data Availability

The data presented in this study are available in this article or supplementary material.

## Supplementary Materials

The following are available online at https://www.mdpi.com/article/10.3390/pharmacy9020081/s1, Supplementary Table S1: Additional exemplar quotes from student reflective journals.
