# Supplementary material for: Experiential Learning in a Gamified Pharmacy Simulation: A Qualitative Exploration Guided by Semantic Analysis
Source: Pharmacy (Basel). 2021 Apr 15;9(2):81. doi: 10.3390/pharmacy9020081 (PMC8167508; doi:10.3390/pharmacy9020081)
Supplement: Supplementary file 1 [file pharmacy-09-00081-s001.zip › pharmacy-1173994-supplementary.pdf]

Supplementary Table S1. Additional exemplar quotes from student reflective journals

Theme	Exemplar Student Quote
**Teamwork**	
Pressure of marks	*However, having the weight of the entire team on my shoulder is a responsibility I do not want to disappoint.* (P004, 2016)<br>*I felt the pressure of being in charge of a pharmacy, but also I felt the pressure of having team mates whose university degree depended on how well we did in the PharmG activity.* (P063, 2017)
Early apprehension	*I was really apprehensive about our team. I did not know if we would mesh well together and it made me nervous that this could impact our ability to provide good patient care and consequently do well in the assessment. … Our team immediately began collaborating and delegating the work load which really motivated me to work together to achieve our objectives. ... The team environment really motivated me through the day.* (P019, 2016)
Positive emotions	*The thing that has been most beneficial in the game was the teamwork. I enjoyed working in a team and feel my skills in this area have improved over the course of the game. We have progressively begun to work more cohesively over the three weeks. The team has been very supportive of all members and everyone has equally taken responsibility for our mistakes*. (P102, 2017)<br>*Being in a team of people where everyone reacts differently to similar situations is very eye opening. The difference of personalities is something that a good manager has to deal with and this is not a small task.* (P003, 2016)<br>*Despite the increasing stress and friction, I feel touched to see how as a team, we often console each other and bring each other up when conquering obstacles together. We also compliment each other when we receive satisfying results. It was rewarding as the simulation brought me closer to certain team members allowing me to gain more respect and trust from them.* (P066, 2017)<br>*This exercise has really made me trust the input of my team.* (P162, 2018)
Insights	*Today I had a reminder to be mindful of the added difficulties that people who don't have English as their first language may experience. I was helping a teammate with a case and was editing their counselling. I felt frustrated and annoyed that they had so many mistakes... It's possible that the mistake was not a careless error but an incorrect word selection due to the fact that the person is struggling with the same content that I am in addition to constantly translating to English. It was a humbling realisation... I think I need to keep this in mind to remind myself to be more patient and to take it into consideration when giving feedback.* (P006, 2016)
**Medicines Provision**	
Positive emotions	*...it was really fun and refreshing to verbally counsel an actual patient. I think what lead into my enjoyment was the fact that I was given time to prepare what I was going to say.* (P017, 2016)
Learning from error	*The case involved an opioid patch, opioid conversion and an overdose. My feelings of joy and satisfaction deflated in an instant as I knew my team did not pick that up. I had some part in that script, and if it was true I would be partially responsible for the potential harm that would be caused to that patient.* (P017, 2016)
Time and workload	*These kinds of difficulties regarding short staffing and busy, stressful days are definitely something that can and will occur in my future career as a pharmacist.* (P082, 2017)
**Patient-Centredness**	
	I felt an overwhelming sense of responsibility and realised we play a huge role in the provision of patient-centered care which involves tailoring patients medication administration and storage to best suit them.

	I am worried I have dispensed medicines in the past and not considered patient-specific factors and hence left patients confused and taking medicines ineffectively. (P005, 2016)
Holistic care	*I discovered that, in an effort to view the patient and their concerns holistically, we must not only apply our medication knowledge to the individual situations but also requires the ability to acquire relevant information by motivating, showing empathy and care towards them.* (P084, 2017)
Reflection for improvement	*There was one situation which took me by surprise. It was in regard to a situation whereby a patient's mother had recently passed and he had brought some medication to be disposed of. I found it difficult to adjust to his emotional status, I felt as if I showed great empathy however I could have been more supportive with my approach.* (P007, 2016)
**Future Practice**	
	I understand that I still have a lot of work to do but now that I have more guidance, I am excited to continue incorporating and practicing holistic care for all patients. I hope that once I am a pharmacist I will continue to practice this skill. (P017, 2016)
	I have been introduced to new resources and ways of accessing information that I believe will truly assist in better delivering patient centred care in the future. Although 'the game' may be over, I now feel more equipped and confident to perform the roles of a pharmacist, as a result of being involved in this immersive learning assessment. (P158, 2018)
Interprofessionalism	*I was humbled that the Doctor wanted to ask for my opinion and let me explain what the issue was. I don't normally expect Doctors to ask me for advice but was pleased that he listen the issue that I had seen, and agreed with my reasoning, and the fact I was able to provide resources for my information. The interaction that I had with him was quite a positive one it has changed my views on how Doctors can behave in a health care team.* (P016, 2016)
**Learning Experience**	
	This experience has actually been so valuable, so much more than lectures or even placement ever could have been. It has completely changed my opinion on community pharmacy. (P025, 2016)
	Going through this experience has made me realise how little thought I had given the practicalities of running a pharmacy. I am a little stressed about the balancing act of running a pharmacy- one that has no monetary risk. I feel like the unexpected workload is an important but sometimes annoying factor that makes the game more like real life. (P017, 2016)
	After experiencing this event, I have learnt a lot more about myself that I didn't realise before, not only has it made me more aware of my skills, but it has improved my confidence. (P062, 2017)
